# The role of intermediaries in connecting individuals to local physical activity – protocol for a pilot feasibility trial

**DOI:** 10.1016/j.conctc.2024.101332

**Published:** 2024-07-09

**Authors:** Megan O'Grady, Deirdre Connolly, Emer Barrett

**Affiliations:** a Discipline of Physiotherapy, Trinity Centre for Health Sciences, St. James' Hospital, D08W9RT, Dublin, Ireland; b Discipline of Occupational Therapy, Trinity Centre for Health Sciences, St. James' Hospital, D08W9RT, Dublin, Ireland

**Keywords:** Exercise, Physical activity, Health promotion, Social prescribing, Link workers, Intermediaries

## Abstract

**Background:**

Intermediaries are health-related workers who facilitate connections to local physical activities. Intermediaries deliver interventions by receiving referrals, conducting assessments, connecting referred individuals to activities and/or services in the community, and following up with them over time. However, it is unclear whether individuals who are referred to physical activities by an intermediary improve their physical activity levels, and what their perspectives and experiences are of participating in this intervention. To date there has been a lack of studies investigating the effect of this intervention on physical activity using appropriate outcome measures.

**Methods:**

This will be a mixed methods pilot feasibility study. Participants will be individuals referred or self-referred to an intermediary and connected to local physical activities. Participants will be recruited through two types of intermediary services in Ireland; social prescribing and local sports partnerships. A total of 30 participants will be recruited (15 per service). Baseline demographic information will be taken upon enrolment to the study and three questionnaires will be completed: the International Physical Activity Questionnaire - Short Form, Self-Efficacy for Exercise Scale and Short Warwick Edinburgh Mental Well-being Scale. The questionnaires will be repeated after 12 weeks and in addition semi-structured interviews will be carried out to explore intervention content and delivery, as well as acceptability of the intervention and evaluation design.

**Discussion:**

This overall aim of this proposed study is to investigate the feasibility of an intervention delivered by an intermediary to improve physical activity and health-related outcomes of community-dwelling individuals.

**Registration:**

ClinicalTrials.gov (NCT06260995).

## Highlights

• Local physical activities can help address the problem of physical inactivity.
• Intermediaries facilitate connections to local physical activities.
• Few studies have investigated feasibility or acceptability of this intervention.
• This study will test feasibility of intermediary interventions to improve health.

## 1 Introduction

Physical inactivity is a major global issue affecting health. Physical activity, sport and exercise resources in the community (local physical activities) offer choice, reduce barriers to participation and improve adherence [[1], [2], [3]]. An ‘intermediary’ is a health-related worker who supports individuals to improve their health and wellbeing. Intermediaries facilitate connections to community resources, services and supports (including local physical activities) to improve health and wellbeing [[4], [5], [6], [7], [8]]. A scoping review of methods used to connect individuals from primary care to local physical activities found strong positive findings for processes that involved referral to an intermediary, when examining the percentage of patients connected with a physical activity opportunity, the percentage of connected patients enrolling in and attending the first session of the physical activity, and acceptability of the intermediary service for primary care professionals and patients [9]. Another rapid scoping review of social prescribing link workers (a type of intermediary) found improvements in physical activity levels and quality of life and/or wellbeing [3].

Intermediaries deliver complex interventions due to the number of components involved, the expertise and skills required by those delivering the intervention, the number of groups targeted and the level of flexibility of the intervention components [10]. When reporting and evaluating complex interventions, it is essential that the contextual factors and causal mechanisms are understood [11]. While interventions are being delivered by intermediaries to facilitate engagement in physical activity, the processes involved in the intermediary ‘intervention’ have been poorly described. For example, the processes of referral/connection to an intermediary, the characteristics of the intermediaries themselves, the profile of individuals referred to an intermediary, the mechanisms of actions/strategies used to connect individuals to local physical activities and the local physical activity opportunities accessed remained unclear [12,13].

To date, the research team have completed a scoping review of the published literature to describe the processes of an intermediary when connecting community-dwelling individuals to local physical activities [14]. This review found many different approaches to establishing this connection, and often the processes of referral, assessment, follow-up, and discharge were poorly described. In order to address these gaps, a qualitative descriptive study was carried out where intermediaries from three different professions across Ireland were interviewed; health promotion and improvement officers, local sports partnership community sports development officers and social prescribing link workers [15]. The aim of this study was to gain insight into the role of intermediaries in Ireland and to further define the processes, practices and procedures in the Irish context.

The two previous studies helped to determine the processes of the ‘intervention’ delivered by an intermediary, i.e., receive a referral or self-referral, conduct an assessment, connect to a local physical activity, followed by a period of follow-up and subsequent discharge from the intermediary service. Based on the results from the scoping review and the qualitative descriptive study, there remained three main evidence gaps: 1) after the individual connects to local physical activities, do they continue to engage in physical activity, 2) what are the perspectives and experiences of individuals referred to an intermediary, and 3) is an intervention delivered by an intermediary effective in improving physical activity and wider health-related outcomes? However, to date there has been a limited number of high-quality studies investigating the effectiveness of the intervention delivered by an intermediary, with only five RCTs identified in the scoping review [[16], [17], [18], [19], [20]].

Effective methods of physical activity promotion are needed, as evidenced by the low numbers of people engaging in sufficient physical activity. Globally, over a quarter of adults are physically inactive [21]. In Ireland, rates of physical inactivity are higher than the global average, with 54–66 % of the population failing to meet physical activity recommendations [22,23]. If the intervention delivered by an intermediary was shown to be effective, it could be a promising method of improving physical activity levels. When evaluating complex interventions, feasibility testing is recommended in order to make decisions about progression to the evaluation phase [10]. Having identified the core elements and contextual factors of interventions delivered by an intermediary in Ireland, the research team are now progressing to the next phase of the study which includes feasibility testing.

A feasibility study asks whether a research study can be done, should we proceed with it, and if so, how [24]. Four feasibility studies were identified in the scoping review, and while all studies found their evaluation designs and interventions to be feasible, none of these studies were carried out in Europe limiting their transferability to the Irish context. The majority focused on clinical populations, and a wide variety of outcome measures were used to assess physical activity and health-related outcomes [[25], [26], [27], [28]]. There is a need therefore to determine appropriate physical activity and health-related outcome measures as well as appropriate evaluation designs relevant to the Irish context and a community-dwelling population, before full-scale evaluations can be carried out. There is also a need to investigate the experiences of those who undergo an intervention delivered by an intermediary. Therefore, the purpose of this proposed study is to examine the feasibility and acceptability of an intervention delivered by an intermediary to improve physical activity levels and wider health-related outcomes, as well as the feasibility of the evaluation design.

## 2 Materials and methods

### 2.1 Intervention

**Fig. 1:**
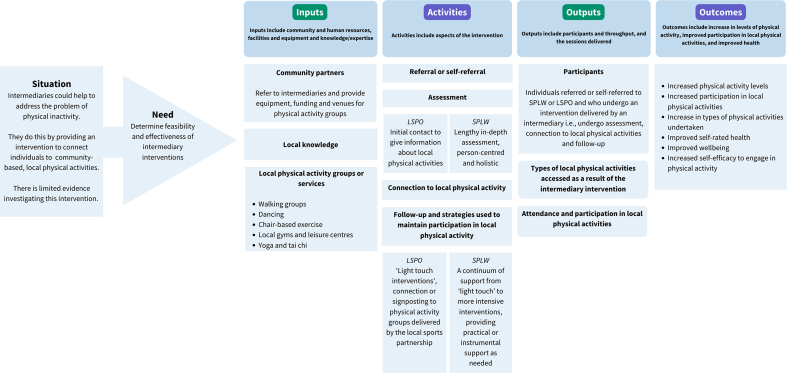
Logic Model Describing an Intervention Delivered by Local Sports Partnership Community Sports Development Officers and Social Prescribing Link Workers in Ireland. Data in this logic model is based on the results of the scoping review and qualitative descriptive study conducted as part of this project [14,15], and is based on Sport England logic model guidance [29]. Abbreviations: LSPO – local sports partnership community sports development officers, PA – physical activity, SPLW - social prescribing link workers.

The intervention that will be examined in this proposed study consists of two phases. The first is the intervention delivered by the intermediary, and the second is attendance and participation in the local physical activity facilitated by the intermediary. The intervention delivered by an intermediary comprises four main steps; referral to an intermediary, assessment of the individual by the intermediary, connection to a local physical activity, and a follow-up period. How the intervention is delivered by the intermediary varies depending on the type of intermediary, the individual who is referred and their needs in relation to their health and wellbeing, the local context and the availability of community services and supports. Logic models were developed based on findings from the scoping review [14] and qualitative study [15] to describe the steps in the intervention delivered by intermediaries (Fig. 1), and are summarised briefly in the following section.

#### 2.1.1 Social prescribing link workers [SPLW]

SPLW receive referrals from healthcare professionals, community services, or through self-referral. Individuals are referred for social isolation, for frequent healthcare attendance, complex social needs, or to address a pre-existing mental or physical health issue [30]. SPLW carry out a lengthy in-depth assessment of the individual's needs in relation to their health and wellbeing, and then work with the individual over a number of weeks and months to connect them to a range of community services and supports, which can include local physical activities. They use a number of strategies during this follow-up period, characterised by ongoing instrumental support (doing things for the individual [31]), empowerment and motivation techniques and/or attending activities with the individual [15].

#### 2.1.2 Local sports partnership community sports development officers [LSPO]

LSPO mainly receive self-referrals, or can receive referrals, to improve the physical activity levels of individuals through participation in physical activity groups delivered by the local sports partnership [32]. LSPO do not carry out an in-depth assessment, but after the referral is received they may carry out screening and explain/signpost to local physical activities. They use a number of strategies to connect the individual to a local physical activity, such as attending the first session of the local physical activity, providing reassurance and practical support to the individual, as well as keeping them updated and informed as to upcoming local physical activities [15].

After the intermediary establishes a connection to a local physical activity, the individual enrols, attends and takes part in the chosen local physical activity/activities. The intermediary continues to support this engagement during the follow-up period. Local physical activities can include walking groups, local gyms and leisure centres, chair-based exercise, outdoor activities (including gardening), yoga and tai chi amongst others.

### 2.2 Aims and objectives

Feasibility is defined as the extent to which a task or innovation can be successfully carried out within a given agency or setting and performed relatively easily or conveniently given existing resources and circumstances [33]. The aim of this proposed study will be to investigate the feasibility of an intervention delivered by an intermediary to improve physical activity and health-related outcomes of community-dwelling adults. Specific objectives include.

i. To determine the rate of recruitment and enrolment to the proposed study of individuals who undergo an intervention delivered by an intermediary, which includes participation in local physical activities (intervention participants).
ii. To examine the suitability and acceptability of recruitment methods to the proposed study.
iii. To determine retention to the proposed study.
iv. To describe the demographic and health characteristics of intervention participants.
v. To determine participation in and adherence to local physical activities by intervention participants.
vi. To explore the acceptability of the intervention delivered by the intermediary.
vii. To explore suitability, acceptability, and completion rates of the measurement tools by intervention participants.
viii. To assess if change occurs in physical activity and health-related outcomes following participation in the intervention.

The CONSORT extension to pilot and feasibility trials, and the SPIRIT guidelines for protocols of interventional trials have been used to guide the reporting of this protocol [[34], [35], [36]].

### 2.3 Research ethics approval

Ethical approval for this proposed study was granted from the Trinity College Dublin Faculty of Health Sciences Research Ethics Committee on August 3, 2023 (Ref: 230603).

### 2.4 Study design

This proposed pilot feasibility study (NCT06260995) will use a sequential explanatory mixed methods study design [37]. This design involves collecting and analysing quantitative data, followed by collection of qualitative data to help explain findings from the quantitative data [37,38]. Findings are then merged in the interpretation or discussion sections [37]. The quantitative phase of the study will be a quasi-experimental before-and-after design (without control) and the qualitative phase will employ a qualitative descriptive approach [39].

### 2.5 Sample

Participants will be those who are referred or self-referred to a social prescribing link worker or local sports partnership community sports development officer and who undergo an intervention delivered by an intermediary i.e., undergo assessment, onward connection to local physical activities and follow-up. A baseline assessment will be taken upon enrolment on to the study, and this assessment will be repeated after 12 weeks from time of enrolment.

### 2.6 Eligibility criteria

Participants will be adults aged >18 years old, able to give explicit informed consent, referred to an intermediary, and that the intermediary facilitates a connection to local physical activities. Inclusion and exclusion criteria are listed in Table 1.

**Table 1:** Study inclusion and exclusion criteria.

Inclusion criteria	• Aged ≥18 years • Referred to intermediary service for any reason OR self-referred to intermediary service for any reason • Meets the eligibility criteria of the intermediary service • Intermediary facilitates a connection to local physical activity
Exclusion criteria	• Non-community-dwelling e.g., hospital in-patients, living in residential care facilities • Diagnosis of dementia • Inappropriate for health or social reasons (such as terminal illness, family or other social crisis) • Refused to engage with the intermediary service • Unable to give explicit informed consent

### 2.7 Sample size

Convenience sampling will be used, which involves selection of participants based on availability and accessibility [40]. The limitations of non-probability sampling techniques are that it may not create a sample that is representative of the population referred to an intermediary, and that it may introduce selection bias [41]. However, convenience sampling is economical, accessible, and has been used in previous feasibility studies investigating intermediaries [26,27]. Therefore, it was selected for this proposed study. The target sample size will be 30 participants, with approximately 15 participants recruited by SPLW and 15 by LSPO. As this proposed study will have three outcome measures (See Section 2.9 ‘Outcomes’), the sample size has been calculated based on the 10 participants per candidate predictor (variable) rule [42,43]. This rule has been used for multivariable methods of analysis, to reduce the risk of unstable risk estimates and misleading associations that could occur with two few or too many events per variable [44].

### 2.8 Recruitment

Nineteen services (N = 11 social prescribing services and N = 8 local sports partnership services), who had taken part in an earlier study as part of this research project, were contacted to act as gatekeepers for this proposed study. Out of nineteen services, N = 11 agreed to act as gatekeepers. Therefore, participants will be recruited from eleven social prescribing (N = 7 services) and local sports partnership (N = 4 services) services from across Ireland, with the goal to recruit up to 3–4 participants from each service. Recruitment will take place over a six-month period, commencing in September 2023. As many of these services do not have access to administrative support, intermediaries usually make their own appointments and/or meet with individuals directly. Therefore, potential participants will be recruited by social prescribing link workers and local sports partnership community sports development officers, who will act as gatekeepers. Study posters will be sent to each service and can be used at the discretion of the gatekeeper e.g., for display in offices or when introducing the study to their clients or to share through mailing lists. Recruitment strategies will vary slightly depending on the category of intermediary, as detailed below.

#### 2.8.1 Social prescribing link workers [SPLW]

If the SPLW plans or makes a connection to, and/or the individual referred expresses an interest in attending, a local physical activity, they are eligible for recruitment and they will be informed about the study. This can occur at the time of referral, the first appointment or subsequent appointments. If the potential participant is agreeable, the SPLW will give them a participant information leaflet and informed consent form (study information) and ask consent to share their contact details with the research team. Following a seven-day period, the research team will then contact those who agree to be contacted to screen for eligibility and obtain verbal and written consent to participate, or participants can contact the research team directly.

#### 2.8.2 Local sports partnership community sports development officers [LSPO]

LSPO meet with individuals for the first time at the initial session of a local sports partnership physical activity programme, or during a screening phone call. Every person that the LSPO meets at these initial sessions or during phone calls during the recruitment period will be informed about the study. If the potential participant is agreeable, the LSPO will then give them the study information and ask consent to share their contact details with the research team. The research team will then contact those who agree to be contacted directly after seven days to screen for eligibility and obtain verbal and written consent to participate, or participants can contact the research team directly.

### 2.9 Outcomes

Definitions of outcomes, measurement tools, and time points for data collection are summarised in Table 2.

**Table 2:** *Data Methods and Schedule of Measures.* Study objectives, descriptions of their associated measures, data measurement tools and the timeframe of data collection. Abbreviations: Short Warwick-Edinburgh Mental Well-being Scale (SWEMWBS), Self-Efficacy for Exercise Scale (SEE), International Physical Activity Questionnaire - Short Form (IPAQ-SF), Numerical Rating Scale (NRS).

Objectives	Measure Definition	Measurement Tool	Data Collection Timeframe		
			Time 0	Time 1 (12 Weeks)	Post-recruitment period
i To determine the rate of recruitment and enrolment to the proposed study of individuals who undergo an intervention delivered by an intermediary, which includes participation in local physical activities (intervention participants).	How many people are approached/receive information about the study from the gatekeeper - # (%) of total clients referred to an intermediary who will be connected to local physical activities i.e., eligible people - # (%) of eligible people who receive study information How many eligible people take part in the study - # (%) eligible people who receive study information who take part in the study	Gatekeeper recruitment log Gatekeeper recruitment log	Ongoing throughout recruitment period Ongoing throughout recruitment period		
ii To examine the suitability and acceptability of recruitment methods to the proposed study.	How long it takes to recruit 3–4 people from each service/gatekeeper - length of recruitment period per service (days) Reasons given for not taking part - frequency count of reasons given for not taking part in research Explore gatekeepers' perceptions of how open clients were to take part in the research, their experience of recruitment and the acceptability of the recruitment methods.	Gatekeeper recruitment log Gatekeeper recruitment log Gatekeeper semi-structured interview items #1-8	Ongoing throughout recruitment period Ongoing throughout recruitment period		**✘**
iii To determine retention to the proposed study.	How many people return for a follow-up assessment with the research team	# (%) of participants who return for 12-week follow-up assessment		**✘**	
iv To describe the demographic and health characteristics of intervention participants.	The age, gender, marital status, urban/rural, level of education, ethnicity, employment status, self-reported presence/absence of chronic condition, self-reported rating of health, and types of exercise currently undertaken of the study sample	Data collection form items #1-10	**✘**	**✘** (Items #9–10 only)	
v To determine participation in and adherence to local physical activities by intervention participants.	Changes in types of exercise undertaken by participants from Week 0 to Week 12 - including self-directed physical activity and/or local physical activities accessed as a result of the intervention	Data collection form item #10 - *“What types of exercise do you do currently? Please provide a list.”* Intervention participant semi-structured interview item #3 - Includes prompts about what local physical activities were accessed as a result of the intervention, and adherence to the local physical activity	**✘**	**✘** **✘**	
vi To explore the acceptability of the intervention delivered by the intermediary.	Explore intervention content and delivery by the intermediary, the impact of the support provided, and how acceptable the intervention was to the participant.	Intervention participant semi-structured interview items #1–6, 11 Intervention acceptability NRS (Intervention participant semi-structured interview item #10)		**✘** **✘**	
vii To explore suitability, acceptability and completion rates of the measurement tools by intervention participants.	Determine if the outcome measures used in this proposed study captured important information/improvements made by the participant, the rates of completion of each outcome measure, and explore experience of completing the outcome measures.	Intervention participant semi-structured interview items #7-9 # (%) items completed in data collection form items #1–10, SWEMWBS, SEE, IPAQ and intervention participant semi-structured interview items #1-11	**✘**	**✘** **✘**	
viii To assess if change occurs in physical activity and health-related outcomes following participation in the intervention.	Change in IPAQ-SF score from Week 0 to Week 12 Change in SEE score from Week 0 to Week 12 Change in SWEMWBS score from Week 0 to Week 12 Changes in self-rated rating of health from Week 0 to Week 12	IPAQ-SF SEE SWEMWBS Data collection form item #9	**✘** **✘** **✘** **✘**	**✘** **✘** **✘** **✘**	

#### 2.9.1 Demographics and health status

Participant age, gender, marital status, area in which they live (urban/rural), level of education, ethnicity, employment status, self-reported presence/absence of chronic condition(s), self-reported rating of health, and types of exercise currently undertaken will be collected at baseline. These data will be used to report demographic information and health-related behaviours of the study sample. A number of questionnaires will be completed at baseline (T0), and again after 12 weeks from the time of the baseline assessment (T1), and these are detailed below. Changes in these scores over time will be examined, and these questionnaires will be tested as suitable outcome measures for future studies investigating effectiveness of intermediaries in improving health- and physical-activity related outcomes over time.

#### 2.9.2 International Physical Activity Questionnaire - Short Form

The International Physical Activity Questionnaire is a self-reported measure of multiple domains of physical activity, such as physical activity undertaken for leisure, work, transport and/or domestic activities [45]. There are two versions, the long form and short form (IPAQ-SF). The original authors of the questionnaire recommend the IPAQ-SF, as it is less burdensome for both participants to complete and researchers to administer. This was an important consideration due to the remote nature of this proposed study (See Section 2.10 ‘Data Collection’). The IPAQ-SF assesses physical activity behaviour over the last seven days and includes measures of frequency and duration of vigorous physical activity, moderate physical activity, walking and sedentary behaviour. Using a standardised scoring protocol, total days of activity, total activity (minutes/week), metabolic equivalent minutes per week and physical activity category (low, moderate or high levels of physical activity) can be calculated [46].

#### 2.9.3 Self-Efficacy for Exercise Scale

The Self-Efficacy for Exercise Scale (SEE) is a self-report measure of self-efficacy for physical activity [47]. This measure was chosen as it measures beliefs about a current capability and is concordant with the physical activity measure used in this proposed study in relation to frequency and duration [48]. Participants are asked to rate their current confidence in their ability to exercise three times per week for 20 min under different scenarios. Responses are rated on a numerical rating scale of 0–10, with 0 indicating ‘not confident’ and 10 indicating ‘very confident’. The overall score is calculated by summing the responses to each scenario, and a higher score indicates higher self-efficacy for exercise.

#### 2.9.4 Short Warwick Edinburgh Mental Well-being Scale

The Warwick-Edinburgh Mental Well-being Scale is used to evaluate interventions, programmes and approaches promoting mental health and wellbeing, particularly in non-clinical populations [49]. This measure was chosen as many individuals who are referred to intermediaries to address loneliness and mental health issues are connected to local physical activities to help address these issues [15]. Physical activity has the potential to improve subjective mood, improve self-esteem and self-perception, cognitive function and sleep quality, all of which may impact on well-being [50]. A seven-item version of the original 14-item scale, the Short Warwick–Edinburgh Mental Well-Being Scale (SWEMWBS), was developed as an interval scale measure of mental well-being [51] and has the added benefit of being shorter and less burdensome to complete. Items cover different aspects of eudemonic and hedonic well-being, and each item is answered on a 1 to 5 Likert scale, ranging from “none of the time” to “all the time” [51]. The overall score is calculated by summing the scores for each item, and a higher score indicates a higher level of well-being.

#### 2.9.5 Semi-structured interviews – intervention participants

**Fig. 2:**
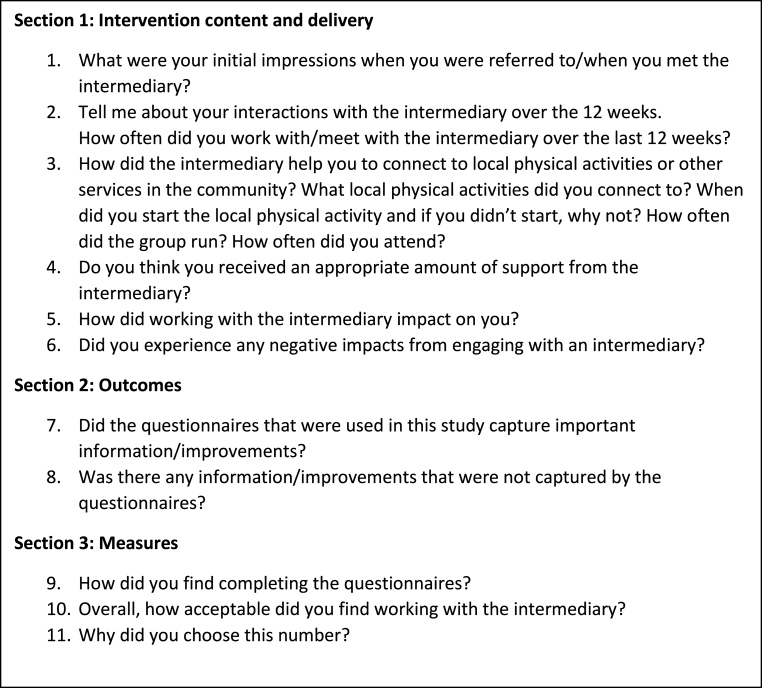
Sample semi-structured interview topic guide for intervention participants.

A semi-structured interview will be completed at the time of the follow-up assessment at 12 weeks with intervention participants, to explore the experiences of those who underwent the intervention (which includes the intervention delivered by the intermediary and attendance and participation in the local physical activity facilitated by the intermediary). A semi-structured approach allows both structure and flexibility, an interactive approach to interviewing, and the use of generative probes, allowing the participant to explore avenues of thought that arise from the interview [52]. This interview will explore topics such as: the content and delivery of the intervention by the intermediary, the local physical activities accessed as a result of the intervention and adherence to these, the experience of engaging in these local physical activities facilitated by the intermediary, the level of support provided by the intermediary and the impact of this, and acceptability of the proposed study measures and intervention by an intermediary. A sample topic guide is outlined in Fig. 2.

#### 2.9.6 Overall acceptability of the intervention

As part of the semi-structured interview, participants will be asked to rate the overall acceptability of the intervention delivered by an intermediary on a numerical rating scale of 1–10, with 1 indicating ‘completely unacceptable’ and 10 indicating ‘completely acceptable’. Assessing acceptability qualitatively and quantitatively is recommended for complex interventions [53]. Including a general acceptability item alongside a qualitative investigation allows researchers to explore what drives participants' general acceptability judgment [54].

#### 2.9.7 Gatekeeper recruitment logs

Gatekeepers will be asked to keep a record of how many clients they had contact with during the recruitment period, how many people were approached about the study, and the reasons for refusal to take part in the study. This record will be kept until 3–4 participants have been recruited from each participating service. For participants recruited to the study, gatekeepers will also be asked to keep a record of their contacts with this person (date, length of each contact and mode of contact). This will help to verify the data collected from participants in their interview.

#### 2.9.8 Semi-structured interviews – gatekeepers

**Fig. 3:**
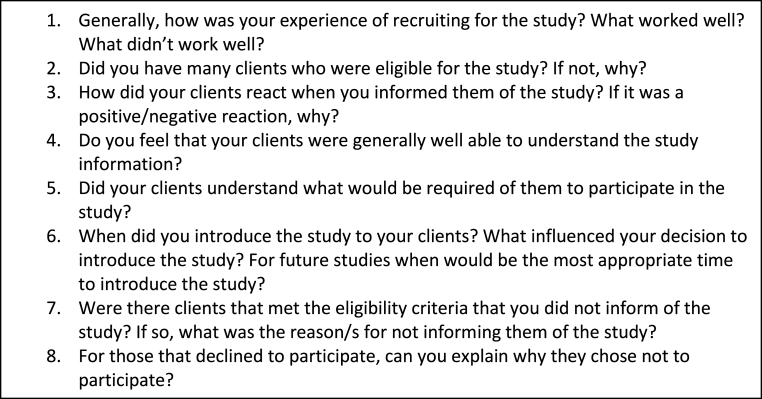
Sample semi-structured interview topic guide for gatekeepers.

In addition to the recruitment logs, semi-structured interviews will also be conducted with gatekeepers to explore the acceptability of the recruitment methods. These interviews will take place at the end of the recruitment period. This interview will explore topics such as: how open individuals were to take part in research and reasons given as to why they did not take part, their experience of discussing the research project with potential participants, and timing of recruitment. A sample topic guide is outlined in Fig. 3.

### 2.10 Data collection

Data will be collected according to the person's preference; online (hosted via Zoom), over telephone, or data collection forms will be posted to participants to complete in their own time (with or without telephone support from the research team).

### 2.11 Data analysis

Quantitative data analysis will be performed using SPSS V28 (IBM, USA) and Microsoft Excel software (Microsoft Corporation, USA). Categorical variables (counts and proportions) and graphical representations e.g., bar charts will be used to present feasibility outcomes. Summary statistics for continuous variables will also be presented as (i) means and standard deviations for normally distributed data, and (ii) median and interquartile ranges for data which was not normally distributed. Within-group differences will be assessed using paired T-tests for normally distributed data and Wilcoxon signed rank tests for data which are not normally distributed.

A qualitative descriptive approach will be taken to the analysis of the exit interviews and free text responses, with the aim of providing a rich, straight description of an experience or an event close to the participant's language [39]. Interviews will be digitally recorded and transcribed verbatim. Transcripts will be analysed using an inductive qualitative content analysis as described by Elo & Kyngäs [55]. Themes will be developed in a four step approach; initialization, construction, rectification and finalization [56]. One researcher [MO’G] will analyse all transcripts using NVivo 14 (QSR International, Australia). The qualitative and quantitative data will then be integrated to provide a comprehensive analysis [37,57].

Progression criteria were not developed for this proposed study, as guidance regarding the application and development of progression criteria for non-randomised feasibility trials is limited [58,59]. In addition, progression criteria are generally used to decide whether to progress a feasibility trial to a definitive controlled trial [60]. It is not yet clear whether a controlled or experimental trial will be the most appropriate methodology for the next phase of this research. By examining feasibility and acceptability of the evaluation design in this proposed study, it is anticipated this will inform future research and trial design.

### 2.12 Data management

Only the research team will have access to data. Detailed descriptions of the plans for data entry, coding, security, confidentiality and storage, including any related processes to promote data quality, are detailed in the Data Protection Impact Assessment created for this proposed study. This has been reviewed and approved by the Trinity College Dublin Data Protection Officer for Research and is available from the authors upon request.

### 2.13 Dissemination

Results from this proposed study will be published as part of a Ph.D. thesis, and in peer-reviewed journals or scientific conferences. The advisory panel associated with this Ph.D. project, consisting of representatives from local sports partnerships, social prescribing link workers and health promotion and improvement officers will be consulted as regards the dissemination plan for this proposed study.

### 2.14 Protocol amendments

Any substantive protocol amendments will be reviewed by the Trinity College Dublin Faculty of Health Sciences Research Ethics Committee, and any deviation from the protocol will be made clear and explained in the final report.

## 3 Discussion

There is a need for community-based interventions to help address the burden of physical inactivity. The scoping review and qualitative descriptive study carried out by the research team identified the processes used by intermediaries to connect individuals to local physical activities. However, it remains unclear how these processes influence physical activity outcomes of clients, and what the experiences are of individuals receiving this intervention. This proposed pilot feasibility study will investigate the feasibility of the intervention delivered by an intermediary, and any indications of change in health- and physical-activity related outcomes. It will also investigate the feasibility and acceptability of the evaluation design, specifically recruitment, retention and choice of outcome measures. This proposed study is timely, as community care services in Ireland are expanded, new chronic disease prevention and management policies and programmes are implemented, and national physical activity guidelines and plans are updated [[61], [62], [63], [64]]. Strengths of this proposed work include the mixed-method design, which we anticipate will generate a rich analysis describing the experiences of intervention participants. An additional strength is the wide variety of recruitment sites, incorporating urban and rural LSPO and SPLW services. This will provide comprehensive feasibility data for a variety of settings with a variety of existing resources. A limitation of the proposed study is its pilot nature, which limits the conclusions that can be drawn about the effectiveness of the intervention. However, understanding factors related to feasibility and acceptability can help to provide information on how an intervention might be replicated or outcomes reproduced in future evaluation trials.

## Funding

The lead author is in receipt of the Glennon Bursary, awarded by the Irish Society of Chartered Physiotherapists, to support this Ph.D. research project for the period January 2023–January 2024. The funding source had no involvement in the protocol design, writing of the report or decision to submit the article for publication.

## CRediT authorship contribution statement

**Megan O'Grady:** Writing – review & editing, Writing – original draft, Visualization, Project administration, Methodology, Funding acquisition, Conceptualization. **Deirdre Connolly:** Writing – review & editing, Writing – original draft, Visualization, Supervision, Project administration, Methodology, Funding acquisition, Conceptualization. **Emer Barrett:** Writing – review & editing, Writing – original draft, Visualization, Supervision, Project administration, Methodology, Funding acquisition, Conceptualization.

## Declaration of competing interest

The authors declare that they have no known competing financial interests or personal relationships that could have appeared to influence the work reported in this paper.

## Data Availability

No data was used for the research described in the article.
